# Using a Residual Pivot Shift as the Indication to Perform a Lateral Extra-articular Tenodesis During ACL Reconstruction Using Autologous Hamstring Grafts Is Associated With Improved Surgical Outcomes: A Retrospective Review of 4755 Cases

**DOI:** 10.1177/03635465251399208

**Published:** 2026-01-16

**Authors:** Mark Porter, Bruce Shadbolt

**Affiliations:** † Canberra Orthopaedics and Sports Medicine, Canberra, ACT, Australia; ‡ Western Sydney University, School of Medicine, Sydney, NSW, Australia; Investigation performed at Barton Private Hospital, Barton, ACT, Australia; and Canberra Private Hospital, Deakin, ACT, Australia

**Keywords:** ACL, reconstruction, recurrence, tenodesis, indications

## Abstract

**Background:**

There is no consensus regarding the appropriate indications for the addition of lateral extra-articular tenodesis (LET) to anterior cruciate ligament (ACL) reconstruction (ACLR).

**Purpose:**

To determine if incomplete correction of the pivot shift during ACLR is an appropriate intraoperative indication to add the LET, in terms of clinical outcome.

**Study Design:**

Cohort study; Level of evidence, 3.

**Methods:**

From January 2018, incomplete correction of the pivot shift relative to the contralateral knee was used as the indication to add LET to ACLR. Patients presenting before this date comprised group A and those after group B. Study criteria included first-ever ACL rupture, participating in pivoting sports, no other significant ligament injury, and surgery within 3 months of injury. Outcomes of interest were recurrent instability, meniscal tears, and patient-reported outcome measures (PROMs): Tegner activity score (TAS), Knee injury and Osteoarthritis Outcome Score subscales for sport and recreation (sport/rec) and knee-related quality of life, subjective International Knee Documentation Committee score, and Lysholm knee score. Statistical analysis was performed.

**Results:**

Group A (2258 patients; 51% male) and group B (2497 patients; 58% male) were similar regarding age, body mass index, knee dominance, time to surgery, graft diameter, and preinjury TAS (*P* > .05). Group A had a lower male-to-female ratio (*P* < .05) and lower posterior tibial slope (*P* = .01). After 2 years, group A had a higher incidence of ACL rerupture than group B (101/2258 [4.5%] vs 75/2497 [3%]; *P* = .01), with a significant sex and group interaction (*P* = .04). Males in group A (4.4%) had a higher rate of recurrence than males in group B (2.4%) at 2 years (*P* = .03), while in females the rate of recurrence was similar (4.5% vs 3.8%; *P* = .42). Group A had a higher incidence of experiencing a subsequent ipsilateral meniscal tear (58/2258 [2.6%] vs 35/2497 [1.4%]; *P* = .02). All PROMs were similar (*P* > .05). Pivot-shift grade before surgery did not correlate with risk of recurrence (*P* = .991). Uncorrected residual pivot shift was associated with a higher ACL graft rupture rate (*P* < .001).

**Conclusion:**

Using incomplete correction of the pivot shift during ACL surgery as the primary indication to perform LET is associated with lower rates of recurrent ACL rupture, and ipsilateral meniscal tears. Pivot-shift grade before surgery was not associated with risk of recurrence, while residual pivot shift after surgery was.

The goal of treating anterior cruciate ligament (ACL) rupture is to restore normal stable function to the knee, and the most pragmatic clinical test available for functional instability is the pivot-shift test, although it is not absolutely reliable.^2,17,18,35^ Persistent rotational instability after ACL reconstruction (ACLR) is one of the main causes of inferior outcomes and recurrent ACL rupture.^2,19,20^ In the presence of an isolated ACL rupture, ACLR can restore stability to the knee.^26,29,30^ However, there is magnetic resonance imaging (MRI) evidence of some damage to the anterolateral complex (ALC) in up to 96% of acute ACL ruptures.^23^ ALC damage is associated with a higher grade of pivot shift when associated with an ACL rupture,^6,17,19,20,39^ and incomplete correction of the pivot shift is associated with an increased risk of recurrent ACL rupture.^11,12^ The most appropriate goal of surgery may be correction of the pivot shift, and failure to achieve this is associated with an increased risk of recurrent ACL rupture.^7,35,38,39^

Over the last 10 years, there has been growing interest in the use of procedures that repair or reconstruct components of the ALC to augment the function of the reconstructed ACL and reduce the risk of recurrent injuries.^7,10,11,25,28,29^ The decision to add a lateral extra-articular tenodesis (LET) is sometimes made before surgery based on both patient and injury factors, rather than on the inadequacy of the ACLR to stabilize the injured knee.^11^ This adjunctive procedure has been shown to be able to improve clinical outcomes after primary and revision ACLR.^35,36^ When the LET technique used in the current study was first described in the literature, the concept of using the presence of a residual asymmetric pivot shift at the time of surgery, immediately after the ACLR, as the primary indication to perform the tenodesis was also introduced.^36^

These previous studies suggest that a residual pivot shift at the time of ACLR may be an appropriate primary indication to add the LET and may select those patients in whom it may be beneficial and thus reduce the risk of recurrent ACL rupture overall.

The goals of the current study were to determine if the practice of adding the LET to knees undergoing an ACLR when there is a residual pivot shift is associated with improved clinical outcomes in terms of recurrent instability, meniscal tears, and patient-reported outcome measures (PROMs); if a high-grade pivot shift in the injured knee before surgery correlated with the risk of ACL graft rupture; and if the presence of a persistent asymmetric pivot shift after ACLR is associated with an increased risk of recurrent ACL rupture.

## Methods

A retrospective review of prospectively collected data from patients satisfying the study criteria detailed in Table 1 was performed. The diagnostic signs for an ACL rupture in all patients were grade 3 on the Lachman test and a pivot shift of at least 1 grade higher than the contralateral uninjured knee. The pivot-shift test was performed in a standard manner and graded as 0 (none), 1 (glide), 2 (clunk), or 3 (gross).^8,15^

**Table 1:** Inclusion and Exclusion Criteria for Patient Recruitment*^a^*

Inclusion Criteria	Exclusion Criteria
Primary ACL rupture—grade 3 on Lachman test with no endpoint, positive pivot-shift test, MRI-diagnosed ACL rupture and confirmed at arthroscopy	Other ligament injury greater than grade 1
	Follow-up data at 2 y postsurgery not complete
Surgery performed within 3 mo of injury	Previous ACL injury in either knee
Single-bundle ACL reconstruction technique using ipsilateral autologous hamstring graft (semitendinosus and gracilis)	Rheumatoid arthritis, connective tissue disease, GLL, or autoimmune disease

a ACL, anterior cruciate ligament; GLL, generalized ligamentous laxity; MRI, magnetic resonance imaging.

All MRI studies were performed on a 3-T magnet MRI machine (Siemens) with standard sequences and images reported on by radiologists with a subspecialist interest in musculoskeletal medicine. The lateral posterior tibial slope (PTS) was also measured as described in the literature by Hudek et al.^16^

On the basis of research published in January 2018, the senior author (M.P.) introduced the policy of adding an LET procedure to an ACLR at the time of surgery if, on reexamination of the knee immediately after completion of the ACL graft fixation, there was a residual pivot shift of at least 1 grade higher than the contralateral uninjured knee. Group A patients were those who presented between January 2013 and December 2017, before the introduction of the use of the LET to correct a residual pivot shift, and group B patients presented after this, between January 2018 and December 2022. No patient in group A had either any form of anterolateral ligament reconstruction or LET performed.

This study was cleared by the research approval authority at our institution.

### Surgical Technique

**Figure 1.**
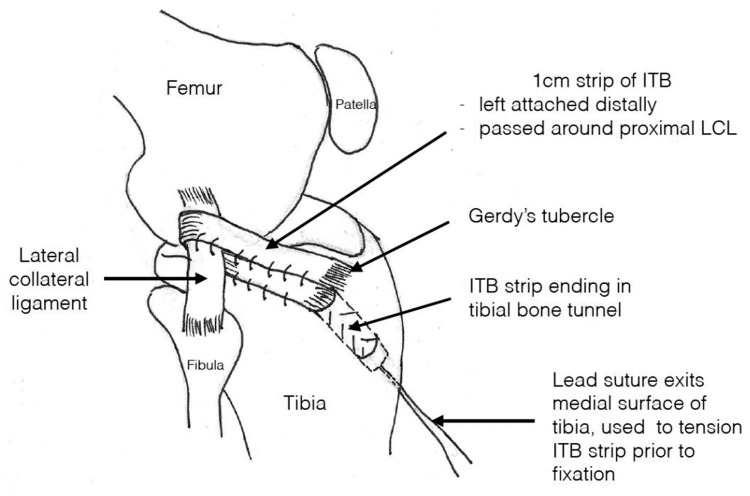
Lateral extra-articular tenodesis technique used by the author. ITB, iliotibial band; LCL, lateral collateral ligament.

Under general anesthesia, both knees were examined and the grade of pivot shifts was recorded for both knees. A standard knee arthroscopy was performed. Any meniscal tear was repaired if possible; otherwise, a partial meniscectomy was performed. After confirmation of ACL rupture, the ACLR was performed using tunnel placement and aperture fixation in a manner shown to maximize control of the pivot shift, but ensuring there was no graft impingement on full extension.^32,33^ The femoral tunnel was drilled using a transportal inside-out technique. Double fixation was used on the femoral side (ACL button, Arthrex; BioRCI HA, Smith & Nephew) and only interference screw fixation on the tibial side (BioRCI HA). The autologous hamstring graft was tripled or quadrupled to produce a graft with a minimum diameter of 8 mm and minimum length of 12 cm. This single-bundle surgical technique used for the ACLR did not change over the study period. After secure graft fixation, the pivot-shift test was repeated, graded, and recorded. In group A, no further surgery was performed on any patient. In group B, if the pivot shift was ≥1 grade higher than the uninjured contralateral knee, the LET was added. The technique used by the author is depicted in Figure 1 and is the same as that described in detail in the literature.^34-36^ An open technique is used to harvest a 15 to 20 cm–long strip of the iliotibial band (ITB), 5 to 10 mm in diameter, left attached at its tibial insertion. It is looped around the proximal lateral collateral ligament from superficial to deep and then passed into a bone tunnel at the tubercle of Gerdy. The lead suture attached to the strip of ITB is passed through the medial tibial cortex near the site of graft harvest, and the tension is adjusted until the pivot shift is the same grade as that of the uninjured knee but without restricting knee movement, before fixation with a third interference screw (BioRCI HA). The defect in the ITB is then closed.

All patients followed a standard physical therapy protocol, which did not change over the study period. Those with no meniscal repair were allowed to bear weight as tolerated after surgery. Patients were evaluated 8 to 12 days postsurgery to check the wounds. Thereafter, a formal standardized physical therapy protocol began, with no twisting or pivoting permitted until at least 6 months postsurgery. Full return to sport was 9 months postsurgery on satisfying all return-to-play criteria. If a meniscal repair was performed, the patient was placed in a brace for 6 weeks postsurgery, with no flexion beyond 90° for 4 weeks, and weightbearing limited to up to 50% body weight for 4 weeks. Thereafter, full flexion and full weightbearing were introduced gradually.

Patients were routinely reviewed at 6 weeks, 6 months, 12 months, and 24 months after surgery, and at any time if they experienced instability, mechanical symptoms, systemic symptoms, or other concerns.

### Outcomes of Interest

#### Primary Outcome Measures

Recurrent ACL rupture was confirmed with both clinical assessment (grade 3 on the Lachman test and a positive pivot-shift test) and repeat MRI studies to reduce potential bias. Meniscal tears were suspected based on clinical features (locking/catching symptoms, effusion, joint line tenderness, and positive McMurray test) and MRI signs of a meniscal tear. A second-look arthroscopy was performed to confirm the diagnoses and then revise the ACLR and/or treat the meniscal injury if required.

#### Secondary Outcome Measures

The following validated PROMs were used, with their minimal clinically important difference in parentheses: Tegner activity score (TAS; 1 point),^4,5^ Knee injury and Osteoarthritis Outcome Score (KOOS) sport and recreation (sport/rec) and knee-related quality of life (QoL) subscales (10 points),^37^ subjective International Knee Documentation Committee (IKDC) score (5 points),^1^ and Lysholm knee score (8.9 points).^4,5^

PROMs were recorded at baseline and 24 months. For those patients who sustained a recurrent ACL injury, the scores were not included in the final analysis, as the precipitous fall in scores after ACL re-reinjury would have biased the outcome against the group with a higher rate of recurrent ACL rupture. Patients completed the PROMs on their own to reduce the risk of bias. The occurrence of any complication related to surgery was also noted.

### Statistical Analysis

The goal of statistical analysis was to test the study’s primary hypothesis that using the presence of a residual pivot shift as the indication to perform the addition of the LET to an ACLR, versus not using this indication, would reduce the risk recurrence of ACL rupture. Secondary outcomes of interest included the influence of the addition of the LET on the risk of contralateral knee injuries (ACL rupture and meniscal tears), and PROMs over a period of 2 years.

The 2 groups were compared regarding baseline characteristics. Categorical data (male-to-female ratios, meniscal tears, limb dominance) were compared using chi-square tests. Continuous variables (age, body mass index [BMI], PTS, and time to surgery) were compared using analysis of variance and generalized linear models. Discrete data (preinjury TAS and ACL graft diameter) were compared using parametric and nonparametric approaches.

In addition to descriptive statistics, the analyses were split into 2 methods. The first approach used individual record data to test differences in PROMs between those in group A and those in group B at baseline and 2 years postsurgery. Multivariate general linear models were used to address multiple testing of the quality of life measures (PROMs) to reduce type 1 error. Baseline and change models at 2 years postsurgery were tested. Models were adjusted for age, sex, BMI, and pivot shift. Analyses of variance of between-patient effects were used in association with the multivariate Wilk lambda.

The second method used aggregated data in weighted generalized linear models with a binary logistic response type to compare the 2 groups. The binary outcome was recurrence of instability within 2 years. Other indicators considered were pivot-shift grade, while adjusting for sex and grade of pivot shift before surgery. The type 3 Wald chi-square test and associated confidence intervals were used for model effects analysis.

The type 3 Wald chi-square test of model effects and cross-tabulation were used to determine if there was difference in the risk of recurrent ACL rupture in those patients within group A with a residual pivot after the ACLR, versus those without.

The type 3 Wald chi-square test of model effects and cross-tabulation were used to determine if there was an association between the grade of pivot shift before surgery and the risk of recurrent instability in both groups A and B, correcting for sex.

Conditional multivariate general linear models were used to compare the groups regarding changes in PROMs over time. The Wilcoxon rank-sum test was used to compare the TAS at 2 years postsurgery.

SPSS Version 29 software was used to conduct the analyses. Two-sided *P* values <.05 were considered significant in support of research hypotheses.

## Results

**Figure 2.**
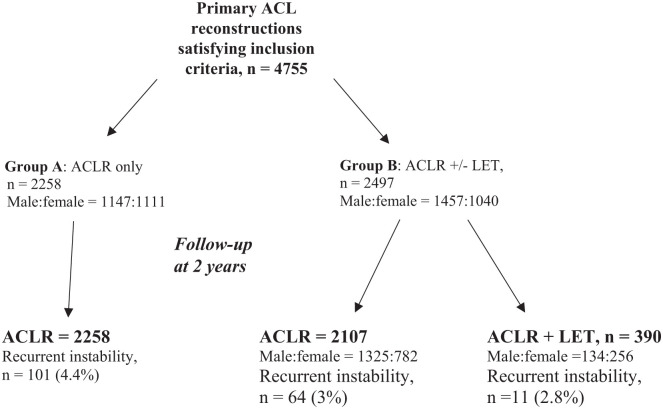
Flow diagram showing patient groups and follow-up. ACL, anterior cruciate ligament; ACLR, anterior cruciate ligament reconstruction; LET, lateral extra-articular tenodesis.

The data from 4755 patients satisfied the inclusion criteria, and Figure 2 is a flowchart following patients through the study. Table 2 details the baseline characteristics of these patients, with the results of statistical analysis comparing the groups.

**Table 2:** Baseline Characteristics of Groups A and B, With Statistical Comparison*^a^*

	Group A	Group B	*P* Value
No. of patients	2258	2497	
Male, %	51	58	**<.05**
Mean age, y	26.4 (7.8)	25.5 (6.1)	.1
Mean BMI	21.5 (1.9)	21.4 (2.3)	.1
Mean PTS, deg	9.6 (1.0)	10 (1.4)	**.01**
Dominant knee, %	45	43	.3
Median TAS preinjury	8 (7-10)	8 (6-10)	.7
Median time to surgery, days	17 (2-42)	18 (2-38)	.6
Median ACL graft size, mm	9 (8-12)	9 (8-11)	.95

a Means are given with SD and medians with range. Boldface type indicates statistical significance. BMI, body mass index; PTS, posterior tibial slope; TAS, Tegner activity score. Group A, patients undergoing ACL reconstruction only; Group B, patients undergoing ACL reconstruction and the addition of LET if indicated.

### Recurrent ACL Rupture and Meniscal Tears

There were 2258 patients in group A (51% male) and 2497 in group B (58% male). The groups were similar regarding age, BMI, baseline TAS, time to surgery, dominance of the injured knee, incidence of meniscal injuries, and graft size (*P* = .1 to .95). The baseline characteristics were recorded on initial presentation to the surgeon and the intraoperative findings at the time of surgery. The baseline TAS referred to the activity level immediately before the knee injury. There was a higher percentage of male patients in group B relative to group A (58% vs 51%; *P* < .05), and group B had a greater mean PTS (10° [SD, 1.4°] vs 9.6° [SD, 1.0°]; *P* < .001) (Table 2).

After adjusting for sex, group A had a significantly higher rate of recurrent ACL rupture than group B at 24 months (101/2258 [4.5%] vs 75/2497 [3%]; *P* = .01). There was a significant sex and group interaction (*P* = .04), with males in group A (4.4%) having a greater rate of recurrence than males in group B (2.4%) at 2 years (*P* = .03), while in females the rate of recurrence in group A (4.5%) was similar to that in group B (3.8%) (*P* = .42) (Table 3).

**Table 3:** Recurrent ACL Ruptures, Contralateral ACL Ruptures, and Meniscal Injuries in Groups A and B at 2 Years Postsurgery*^a^*

	Group A	Group B	*P* Value
No. of patients	2258	2497	
Male, %	51	58	
Recurrent ACL rupture	101 (4.5)	75 (3.0)	**.01**
Male	51 (4.4)	35 (2.4)	**.03**
Female	50 (4.5)	40 (3.8)	.42
Ipsilateral meniscal tear	58 (2.6)	35 (1.4)	**.02**
Male	31 (2.7)	19 (1.3)	**.01**
Female	27 (2.4)	16 (1.5)	.13
Contralateral ACL rupture	106 (4.6)	123 (4.9)	.63
Male	52 (4.5)	59 (4.1)	.53
Female	54 (4.9)	64 (6.1)	.31
Contralateral meniscal tear	16 (0.7)	14 (0.6)	.37
Male	9 (0.8)	8 (0.6)	.34
Female	7 (0.6)	6 (0.6)	.75

a Data are presented as n (%) unless otherwise indicated. Boldface type indicates statistical significance. ACL, anterior cruciate ligament.

After adjusting for sex, group A had a higher rate of subsequent meniscal tears at 24 months relative to group B (58/2258 [2.6%] vs 35/2497 [1.4%]; *P* = .02). The number of meniscal tears was too small to allow medial and lateral meniscal tears to be analyzed separately (Table 3).

After adjusting for sex, group A was not significantly different in the rate of either contralateral ACL rupture (4.6% vs 4.9%; *P* = .63) or contralateral meniscal tears (0.7% vs 0.6%; *P* = .37) relative to group B at 24 months.

There was a significantly higher risk of recurrent ACL rupture in those patients within group A with a residual pivot after the ACLR versus those without (11% vs 3.5%; *P* < .001), and this was independent of sex (Table 4). In both groups A and B, the grade of pivot shift before surgery was not associated with the risk of recurrent ACL rupture (*P* = .991). However, on subgroup analysis, males in group A with a grade 3 pivot shift before surgery did have an increased risk of recurrence (6/101; 6%) relative to those with a grade 1 (25/590; 4.2%) or grade 2 (20/456; 4.4%) pivot shift (*P* = .04) (Table 5). This was not seen in group B or when groups A and B were combined. Within group B, the rate of recurrent ACL rupture was similar in patients with or without the addition of the LET (*P* = .83) (Table 6).

**Table 4:** Group A Patients Subgrouped Into Patients With and Without an Uncorrected Residual Pivot Shift After Their ACL Reconstruction at the Time of Surgery*^a^*

	No Pivot Shift	Residual Pivot Shift	Total	*P* Value
No. of patients	1977 (88)	281 (12)	2258 (100)	—
Male	1021	126	1147	—
Female	956	155	1111	—
Recurrence	70 (3.5)	31 (11)	101 (4.5)	**<.001**
Male	34 (3.3)	15 (12)	49 (4.3)	**<.001**
Female	36 (3.8)	16 (10)	52 (4.7)	**<.001**

a Data are presented as n (%) unless otherwise indicated. Boldface type indicates statistical significance. ACL, anterior cruciate ligament.

**Table 5:** Incidence of Recurrent ACL Rupture by Grade of Pivot Shift Before Surgery in Groups A and B*^a^*

Pivot-Shift Grade	Recurrence	Stable	Total	Recurrence, %	*P* Value
Group A					
Male					
1	25	565	590	4.2	.08
2	20	436	456	4.4	.16
3	6	95	101	5.9	.04
Total	51	1096	1147	4.4	**.005**
Female					
1	16	341	357	4.5	.62
2	24	508	532	4.5	.99
3	10	212	222	4.5	.84
Total	50	1061	1111	4.5	.46
Male and female					
1	41	906	947	4.3	.89
2	44	944	988	4.5	.89
3	16	307	323	5.0	.63
Total	101	2157	2258	4.5	**.009**
Group B					
Male					
1	15	615	630	2.4	.08
2	17	616	633	2.7	.16
3	3	191	194	1.5	.04
Total	35	1422	1457	2.4	**.005**
Female					
1	16	405	421	3.8	.62
2	16	406	422	3.8	.60
3	8	189	197	4.1	.84
Total	40	1000	1040	3.8	.46
Male and female					
1	31	1020	1051	2.9	.11
2	33	1022	1055	3.1	.81
3	11	380	391	2.8	.91
Total	75	2422	2497	3.0	**.009**

a Data are presented as number of patients unless otherwise indicated. Boldface type indicates statistical significance. ACL, anterior cruciate ligament.

**Table 6:** Group B Patients With and Without the LET, Comparison of Recurrence Rates, and Meniscal Tears*^a^*

	ACL	ACL + LET	Total	*P* Value
No. of patients	2107 (84)	390 (16)	2497 (100)	
Male	1325 (63)	134 (34)	1459 (58)	
Female	782 (37)	256 (66)	1038 (42)	
Recurrence	64 (3.0)	11 (2.8)	75 (3)	.83
Male	31 (2.3)	4 (3.0)	35 (2.4)	.61
Female	33 (4.2)	7 (2.7)	40 (3.8)	.28

a Data are presented as n (%) unless otherwise indicated. ACL, anterior cruciate ligament; LET, lateral extra-articular tenodesis.

There were no patients in whom the clinical and MRI diagnosis of recurrent ACL did not correlate with arthroscopic findings.

The risk of recurrence in the subgroups with or without a residual pivot shift within group A were compared using a 2-sided chi-square test for all patients, male patients, and female patients.

### Patient-Reported Outcome Measures

The patients in both groups A and B had significant improvements in all PROMs between baseline at 2 years after surgery (*P* < .001). In group A, the IKDC score was 51.4 (SD, 6.5) at baseline versus 90 (SD, 6.3) at 2 years; KOOS sport/rec, 60.1 (SD, 6.7) versus 87.8 (SD, 8.1); KOOS QoL, 58.5 (SD, 8.0) versus 91.8 (SD, 4.3); and Lysholm knee score, 56.4 (6.3) versus 93.0 (4.4). In group B, the IKDC score was 51.3 (SD, 6.5) versus 89.0 (SD, 6.7), KOOS sport/rec, 61.0 (SD, 6.6) versus 87.1 (SD, 7.6); KOOS QoL, 57.8 (SD, 8.1) versus 92 (SD, 4.7); and Lysholm knee score, 57 (SD, 6.6) versus 94 (SD, 4.9). There were no differences between the groups regarding improvement in scores over time (*P* = .91-.96). TAS values at 2 years were also similar in both groups at 2 years (group A: median, 8 [range, 6-10] vs group B: median, 8 [range, 6-10]; *P* = .95).

### Complications

Septic arthritis occurred in 1 patient in group A and 2 patients in group B, and all were treated with arthroscopic lavage and debridement, in conjunction with 48 hours of intravenous antibiotics and then oral antibiotics for 6 weeks. None of these patients had an LET performed, and the ACL graft was preserved in all cases. Group A had 3 cases of arthrofibrosis and 1 case of cyclops lesion, and group B had 1 case of arthrofibrosis and 1 case of cyclops lesion, all of which were managed with arthroscopic debridement and made a complete recovery.

## Discussion

The main finding in this study was that using the presence of a residual asymmetric pivot shift found on intraoperative reexamination of the knee immediately after the performance of an ACLR as the indication to add an LET at the time of surgery is associated with a significant reduction in recurrent ACL rupture in the population of patients undergoing ACLR when followed up for 2 years. The reduction in recurrent ACL rupture was more significant in males but not in females, when sex-specific analyses were performed.

The LET was indicated in 16% of all patients evaluated with an ACL injury. The grade of pivot shift before surgery was not associated with the risk of recurrent ACL rupture, while the presence of a residual asymmetric pivot shift left uncorrected after ACLR was associated with a higher risk of recurrence. Males with a high-grade pivot shift before surgery may be a subgroup most likely to benefit from augmentation of the ACLR with the LET. The risk of recurrent ACL rupture was similar in those patients with and without the addition of the LET, if the pivot shift was corrected. The use of the presence of a residual asymmetric pivot shift as the indication to add the LET did not significantly influence any of the PROMs, nor was it associated with an increased risk of complications.

Although multiple factors influence the decision to return to sport, there is a large variation in success rates after ACLR, and 50% to 90% patients return to their previous level of sports participation after an ACLR.^9,34-36^ Supplementary LET procedures are not required for most patients undergoing ACLR but may be indicated in some. The indiscriminate use of LET may not be cost-effective, and the use of a staple for femoral fixation with some LET techniques is associated with an increased risk of hardware irritation and/or damage to the femoral tunnel necessitating staple removal.^11,27^

Even in the presence a grade 3 pivot shift, laboratory studies have shown that isolated ACLR can correct the pivot shift,^30^ but when there is significant damage to the ALC of the knee, an isolated ACLR is not able to restore rotational stability, while the addition of an ALC reconstructive procedure is.^3,10,28,29,31^

In this study, we found that one group most likely to benefit from the use of an LET may be males with a high grade of pivot shift before surgery. Multiple indications have been proposed for the addition of an ALC procedure to ACLR, including female athletes, soccer players, elite athletes, inherent ligamentous laxity, increased PTS, high-grade pivot shift, revision ACLR, meniscal root tears, complete or subtotal meniscectomy, and chronic ACL deficiency.^3,12,22^ Many of these indications are based on patient and injury factors and the decision made before surgery, rather than on the effectiveness of the ACLR performed in restoring stability in that particular knee. Laboratory research has shown that when the ACL injury is isolated, an appropriately performed ACLR can restore stability even in the presence of a grade 3 pivot shift in vitro.^30^ Often, injury to the ACL is not isolated, with some damage to the ALC being present in most cases.^23^ The presence of damage to components of the ALC has been associated with an inferior outcome if an isolated ACLR is performed.^39^ Meniscal tears may also contribute to the severity of the anterolateral rotatory instability (ALRI) and thus the grade of pivot.^17^ There may be >1 factor contributing to the ALRI, and some of these factors may heal naturally.^21^ The complex interaction of these various contributory factors is not fully understood, but their combined influence may manifest in the severity of the ALRI and thus the grade of pivot shift.^40,41^ The grade of pivot shift correlates well with functional outcome after surgical stabilization and remains the most practical measure of ALRI.^2,8,20,24^ For this reason, we believe that correction of the pivot shift is the most appropriate intraoperative measure of technical success after surgical stabilization of the ACL-injured knee. Research has found that both anterolateral ligament reconstruction and LET are effective in improving the outcomes after ACLR, likely due to their similar effect in improving rotational stability.^28^

The STABILITY study found that the use of an LET can reduce the risk of clinical failure after ACLR from 40% to 25% in patients they defined as having a high risk of recurrent ACL rupture on the basis of having at least 2 of the following 3 criteria: 1) participation in competitive pivoting sports, 2) presence of grade 2 pivot shift or greater, and/or 3) generalized ligamentous laxity (Beighton score of at least 4) or genu recurvatum >10°.^11^ The 40% failure rate within 2 years in their study is much higher than that in our highest-risk subgroup undergoing isolated ACLR, which was 12% over 2 years in males with a residual pivot shift. Their failure rate of 25% in those patients undergoing ACLR + LET is also much higher than the highest failure rate in our subgroup, with our highest failure rate after ACLR + LET being 3.0% over 2 years. The lower failure rates we found may reflect the fact that their study involved multiple surgeons, with variable levels of technical proficiency and experience. The femoral fixation used in the STABILITY study was suspensory fixation, while the technique used in the current study added femoral interference screw fixation. The latter has been shown in a single in vivo computer navigation study to improve rotational control relative to suspensory fixation.^33^ It is possible that the LET procedure may contribute more to knee stability in the setting of an ACLR, which is performed in a manner that does not maximally control the pivot shift. It is of interest that the STABILITY study defined failure of ACLR as the presence of a positive pivot shift in the follow-up period, and our study supports the use of incomplete correction of the pivot shift at the time of surgery as the indication to add the LET to correct this residual pivot shift when present. Also of interest, Helito et al^14^ performed a study in which they added an LET procedure to knees with a degree of ongoing instability, defined as the presence of a positive pivot shift, and found that this indication to add the ALC procedure was associated with improved outcomes and reduced risk of recurrence. These latter studies would seem to be using similar indications to ours, except we apply the indication during the primary ACLR procedure, rather than at a later date.

Another factor that may contribute to lower risk of recurrence in our study may be the LET technique used. The LET we describe is designed to restore the sheetlike function to the ALC complex, as described by Guenther et al,^13^ and the final tension in the graft is adjusted to the point that the pivot shift test is similar to that on the nonoperative side. This may be preferable to the use of “minimal tension applied to the graft with the knee at 60-70 degrees of flexion and neutral rotation,” as described in the STABILITY study.^11^

The current study does have limitations. Although the data were collected prospectively, the study is a retrospective review of the data, reflecting the sequence of events that should occur in clinical practice after the adoption of a new indication. It is possible that there may be confounding variables within the groups. The groups were sequential, but the surgeon had performed >5000 ACLRs before the commencement of the study and was well advanced along the learning curve using a technique that did not alter over the study. The groups were not compared with regard to the presence of meniscal tears and their management, smoking or nicotine use, and the precise time between surgery and their return to sport, and these remain potential confounding factors. The study findings may have limited external validity and may only be applicable to orthopaedic surgeons with a similar level of experience who subspecialize in soft tissue knee reconstructions. We have assumed that the pivot shift in the operated knee before injury was the same as that in the uninjured contralateral knee. Diagnostic criteria for recurrent ACL rupture and meniscal tears included both clinical signs and MRI findings, which would reduce any potential bias when the unblinded author (M.P.) was examining patients. The radiologists reporting on the MRI studies were not involved in the conduction of the study but could not be blinded regarding the performance of an LET.

We have found that the use of a residual asymmetric pivot shift as the indication to add the LET to an ACLR is associated with a reduced risk of both recurrent ACL rupture and meniscal tears in a population of patients presenting with ACL rupture. The grade of pivot shift before ACLR was not associated with the risk of recurrent ACL rupture, while incomplete correction of an asymmetric pivot shift was.
